# Comprehensive analysis of the correlation of the pan-cancer gene HAUS5 with prognosis and immune infiltration in liver cancer

**DOI:** 10.1038/s41598-023-28653-6

**Published:** 2023-02-10

**Authors:** Wenbing Zhang, Chi Yang, Yan Hu, Ke Yi, Wangwen Xiao, Xiaohui Xu, Zhihua Chen

**Affiliations:** 1 Suzhou Medical College of Soochow University, Suzhou, Jiangsu China; 2 Department of General Surgery, The First People’s Hospital of Taicang, Taicang Affiliated Hospital of Soochow University, No. 58 Changsheng South Road, Taicang, Suzhou, 215400 Jiangsu People’s Republic of China; 3 Central Laboratory, The First People’s Hospital of Taicang, Taicang Affiliated Hospital of Soochow University, No. 58 Changsheng South Road, Taicang, Suzhou, 215400 Jiangsu People’s Republic of China

**Keywords:** Cancer, Cell division, Cell growth

## Abstract

Liver hepatocellular carcinoma (LIHC) is one of the most common malignancies and places a heavy burden on patients worldwide. HAUS augmin-like complex subunit 5 (HAUS5) is involved in the occurrence and development of various cancers. However, the functional role and significance of HAUS5 in LIHC remain unclear. The Cancer Genome Atlas (TCGA), Genotype-Tissue Expression (GTEx), Cancer Cell Line Encyclopedia (CCLE) and Gene Expression Omnibus (GEO) databases were used to analyze the mRNA expression of HAUS5. The value of HAUS5 in predicting LIHC prognosis and the relationship between HAUS5 and clinicopathological features were assessed by the Kaplan–Meier plotter and UALCAN databases. Functional enrichment analyses and nomogram prediction model construction were performed with the R packages. The LinkedOmics database was searched to reveal co-expressed genes associated with HAUS5. The relationship between HAUS5 expression and immune infiltration was explored by searching the TISIDB database and single-sample gene set enrichment analysis (ssGSEA). The Clinical Proteomic Tumor Analysis Consortium (CPTAC) and the Human Protein Atlas (HPA) databases were used to evaluate HAUS5 protein expression. Finally, the effect of HAUS5 on the proliferation of hepatoma cells was verified by CCK-8, colony formation and EdU assays. HAUS5 is aberrantly expressed and associated with a poor prognosis in most tumors, including LIHC. The expression of HAUS5 is significantly correlated with clinicopathological indicators in patients with LIHC. Functional enrichment analysis showed that HAUS5 was closely related to DNA replication, cell cycle and p53 signaling pathway. HAUS5 may serve as an independent risk factor for LIHC prognosis. The nomogram based on HAUS5 had area under the curve (AUC) values of 0.74 and 0.77 for predicting the 3-year and 5-year overall survival (OS) of LIHC patients. Immune correlation analysis showed that HAUS5 was significantly associated with immune infiltration. Finally, the results of in vitro experiments showed that when HAUS5 was knocked down, the proliferation of hepatoma cells was significantly decreased. The pan-oncogene HAUS5 is a positive regulator of LIHC progression and is closely associated with a poor prognosis in LIHC. Moreover, HAUS5 is involved in immune infiltration in LIHC. HAUS5 may be a new prognostic marker and therapeutic target for LIHC patients.

## Introduction

Liver cancer is one of the most common causes of cancer-related death worldwide which remains a global health challenge, and its incidence is growing worldwide^1^. Hepatocellular carcinoma (HCC) is the most common type of primary liver cancer, accounting for approximately 80% of all cases^2^. Hepatitis B and C virus infection, alcohol abuse and ingestion of the fungal metabolite aflatoxin B1 are the major risk factors of HCC^3^. Hepatic resection is the treatment of choice for hepatocellular carcinoma in patients without cirrhosis^4^. However, the high recurrence rate of patients even treated in the early stage and the low survival rate of patients in advanced stage have become the main problems in the treatment of liver cancer^5^. Due to the poor prognosis after treatment, immunotherapy is being intensively studied as an additional treatment and the combination of the anti-PDL1 antibody atezolizumab and the VEGF-neutralizing antibody bevacizumab has or will soon become the best available first-line treatment for advanced HCC^6,7^. Tumor growth markers can be used for targeted prevention of tumor progression^8,9^. However, in the face of economic challenges, drug resistance and efficacy issues, there is still much uncertainty regarding targeted therapies^10^. Therefore, there is an urgent need to search for new potential markers for the diagnosis and treatment of liver cancer.

HAUS augmin-like complex subunit 5 (HAUS5), also known as Dgt5 or KIAA0841, is a member of the HAUS family, which localized to interphase centrosomes and mitotic spindle microtubules. HAUS5 is part of the HAUS augmin-like complex, a microtubule-binding complex first identified in Drosophila, which contributes to mitotic spindle assembly, maintains centrosome integrity and completes cellular dynamics^11,12^. Originally, the outer γ-tubulin ring complex (γ-TuRC) subunits (Dgrip71, 75, 128, and 163) and 5 augmin subunits (called Dgt2–6) are necessary for localizing γ-tubulin to spindle microtubules but not to the centrosomes^13^. The microtubule-associated protein EML3 regulates mitotic spindle assembly by recruiting the augmin complex to spindle microtubules, and the octameric protein complex augmin recruits the γ-TuRC to facilitate robust spindle assembly^14,15^. Although it is known the augmin complex plays an important role in the correct assembly of the spindle, few studies have been conducted on HAUS5. A recent study suggested that HAUS5 is a potential biomarker in breast cancer^16^. However, the role of HAUS5 in hepatocellular carcinoma remains unknown and deserves further investigation.

In this study, we evaluated the expression and prognostic value of HAUS5 in various tumors and assessed its correlation with clinical characteristics in LIHC based on TCGA and multiple public databases. We studied the biological pathways related to HAUS5 using Gene Ontology (GO) and Kyoto Encyclopedia of Genes and Genomes (KEGG) pathway enrichment analysis and GSEA. A nomogram was constructed to predict 3- and 5-year survival rates for patients with liver cancer. We further investigated the co-expressed genes as well as mutations in all patients with hepatocellular carcinoma and the correlation between HAUS5 expression and immune infiltrates. In vitro, knockdown of HAUS5 inhibited the proliferation of hepatoma cells, highlighting the potential carcinogenic role of HAUS5 in liver cancer. Our results suggested that HAUS5 could be a promising prognostic biomarker and therapeutic target for LIHC patients.

## Results

### Significant differences in HAUS5 expression and assessment of HAUS5 As a pan-cancer prognostic biomarker

**Figure 1:**
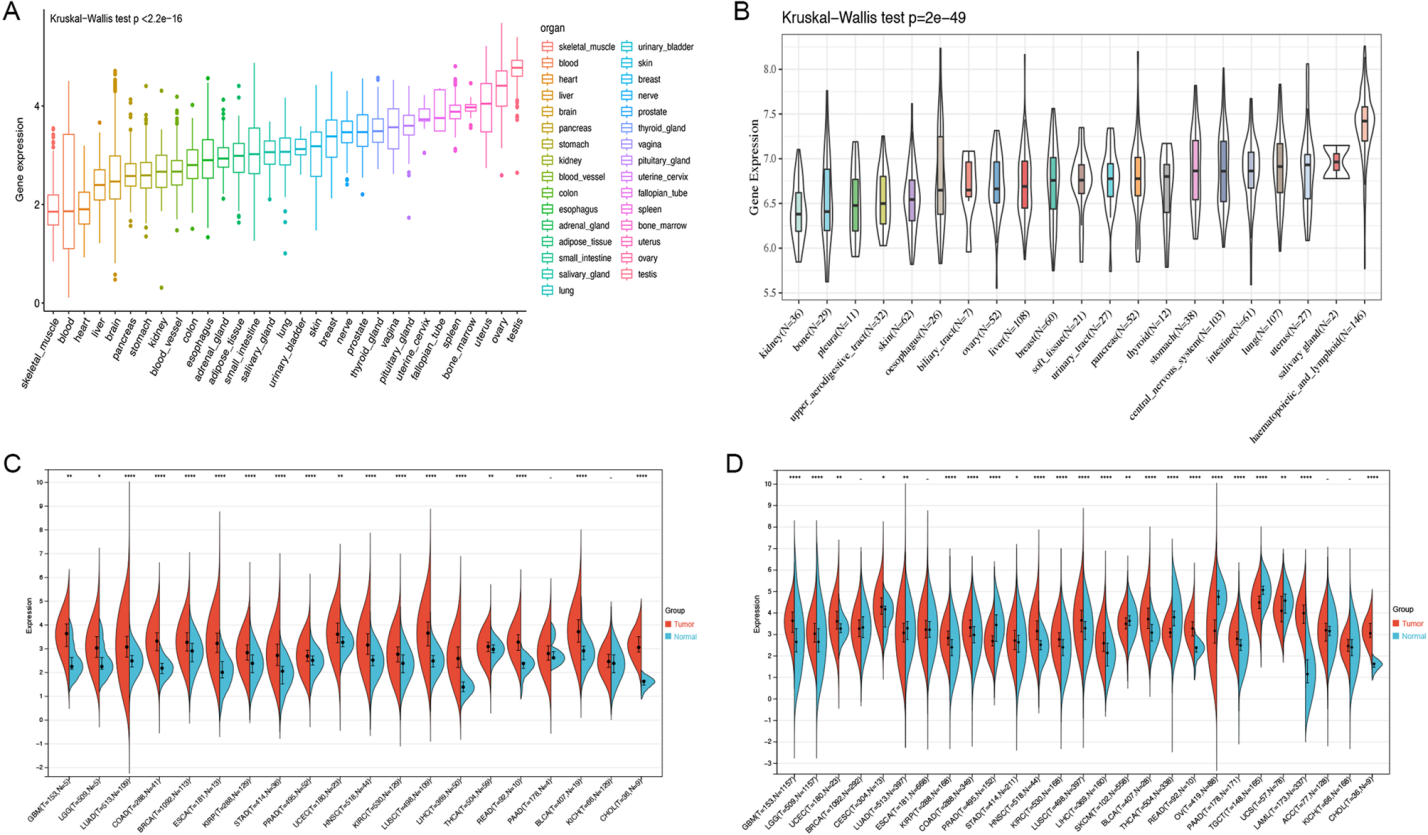
Differential expression of HAUS5. (**A**) HAUS5 expression in normal tissues from the GTEx database. (**B**) HAUS5 expression in tumor cell lines based on the CCLE database. (**C**) HAUS5 expression in 20 types of cancer in the TCGA database. (**D**) HAUS5 expression between tumor and normal samples from the TCGA and GTEx databases. *P < 0.05, **P < 0.01, ***P < 0.001, ****P < 0.0001.

We analyzed the HAUS5 expression level in normal tissues using the GTEx dataset. As shown in Fig. 1A, compared with other tissues, the expression levels were relatively high in ovary and testis tissues and relatively low in blood and liver tissues. We downloaded data of tumor cell lines from the CCLE database and analyzed the expression of HAUS5 in multiple tumor cells (Fig. 1B). From the result, it was observed that the expression level of HAUS5 was generally elevated and significantly different in cancer cell lines (Kruskal Wallis test P = 2e−49) of different tissue origins, which was consistent with the analysis of TCGA database that most tumor tissues expressed higher than normal tissues. To further determine the differential expression of HAUS5, we analyzed HAUS5 mRNA levels in 20 different tumors and normal tissues. As shown in Fig. 1C, HAUS5 mRNA levels were significantly higher in glioblastoma (GBM)^17^, brain lower grade glioma (LGG), lung adenocarcinoma (LUAD), colon adenocarcinoma (COAD), breast invasive carcinoma (BRCA)^16^, esophageal carcinoma (ESCA), kidney renal papillary cell carcinoma (KIRP), stomach adenocarcinoma (STAD), prostate adenocarcinoma (PRAD), uterine corpus endometrial carcinoma (UCEC), head and neck squamous cell carcinoma (HNSC), kidney renal clear cell carcinoma (KIRC), lung squamous cell carcinoma (LUSC), LIHC, thyroid carcinoma (THCA), rectum adenocarcinoma (READ), bladder urothelial carcinoma (BLCA) and cholangiocarcinoma (CHOL) tissues than in normal tissues. Due to the small normal sample size of some cancers, we integrated the GTEx and TCGA datasets with log2(x + 1) expression value and analyzed the differences of HAUS5 expression in 27 cancer types. We found high expression of HAUS5 in tumors, including GBM, LGG, UCEC, cervical squamous cell carcinoma (CESC), KIRP, COAD, STAD, HNSC, KIRC, LUSC, LIHC, BLCA, READ, pancreatic adenocarcinoma (PAAD), acute myeloid leukemia (LAML) and CHOL, but lower than normal expression in LUAD, PRAD, skin cutaneous melanoma (SKCM), THCA, ovarian serous cystadenocarcinoma (OV), testicular germ cell tumors (TGCT) and uterine carcinosarcoma (UCS) (Fig. 1D).

**Figure 2:**
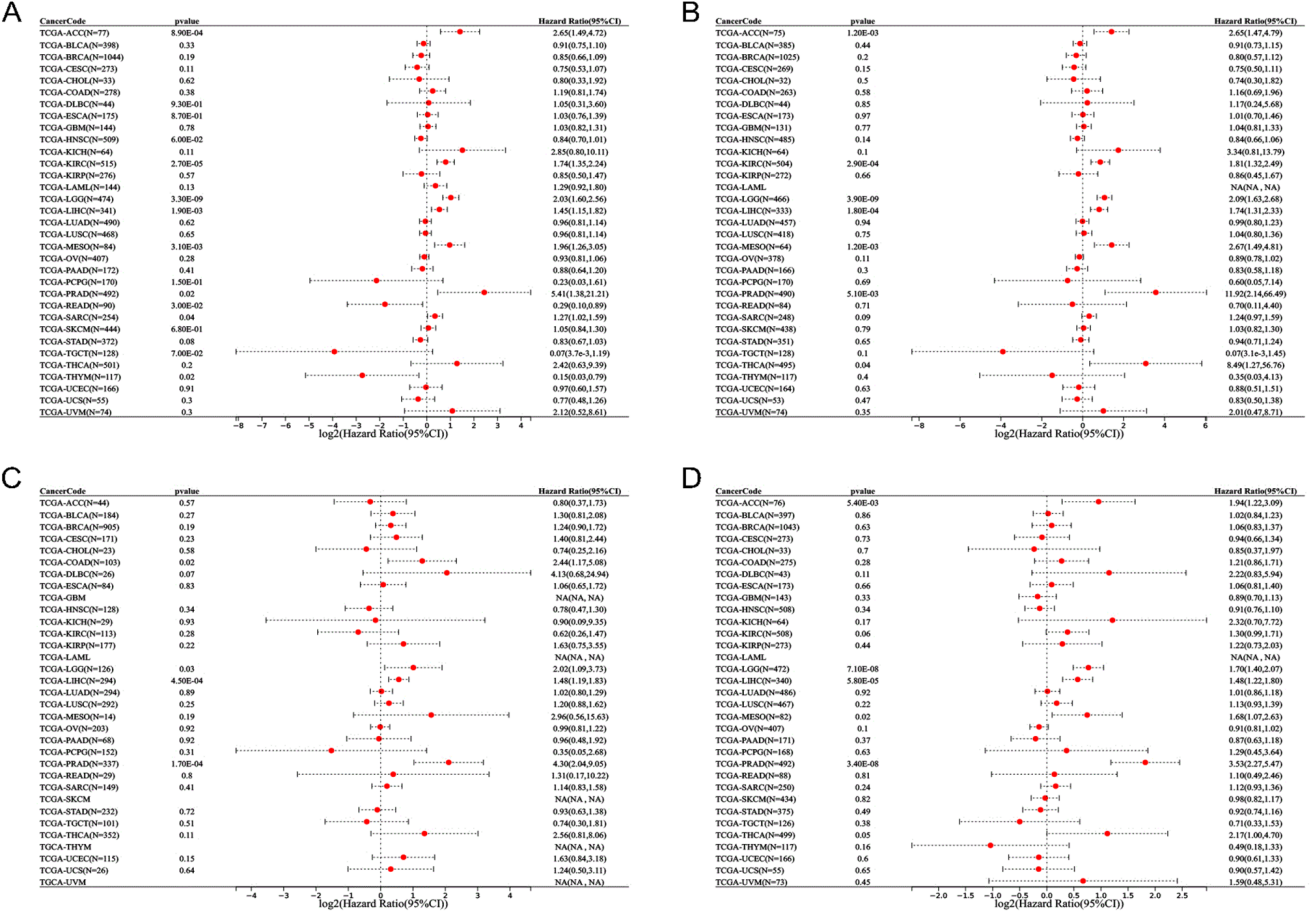
Forest plots of survival analysis based on HAUS5 expression in 33 types of tumors based on the SangerBox database. (**A**) ACC, KIRC, LGG, LIHC, MESO, PRAD, READ, SARC and THYM patient OS showed a significant correlation with HAUS5 expression. (**B**) HAUS5 expression is significantly related to DSS in ACC, KIRC, LGG, LIHC, MESO, PRAD and THCA. (**C**) The forest plot indicates that COAD, LGG, LIHC and PRAD patient DFI values are significantly related to the expression of HAUS5. (**D**) ACC, LGG, LIHC, MESO and PRAD patient PFI values are significantly related to HAUS5 expression. OS, overall survival; DFI, disease-free interval; DSS, disease-specific survival; PFI, progression-free interval.

After confirming that HAUS5 was overexpressed in most tumors, we further explored whether HAUS5 expression was associated with prognosis in pan-cancer patients. We analyzed the relationships between gene expression and prognosis in 33 tumors. From Fig. 2A, we found that HAUS5 expression significantly affected OS in patients with multiple cancer types, including adrenocortical carcinoma (ACC) (p = 8.90E−04), KIRC (p = 2.70E−05), LGG (p = 3.30E−09), LIHC (p = 1.90E−03), mesothelioma (MESO) (p = 3.10E−03), PRAD (p = 0.02), READ (p = 3.00E−02), sarcoma (SARC) (p = 0.04) and thymoma (THYM) (p = 0.02). Furthermore, HAUS5 was a low-risk gene in READ and THYM, while it was a high-risk gene in other types of cancer. Taking into account the possible presence of nontumor mortality factors during follow-up, we analyzed the relationship between HAUS5 expression and disease-specific survival (DSS). In Fig. 2B, high HAUS5 expression was associated with poor prognosis in patients with ACC (p = 1.20E−03), KIRC (p = 2.90E−04), LGG (p = 3.90E−09), LIHC (p = 1.80E−04), mesothelioma (MESO) (p = 1.20E−03), PRAD (p = 5.10E−03) and THCA (p = 0.04). In terms of associations between HAUS5 expression and disease-free interval (DFI), the forest plot (Fig. 2C) showed a poor prognosis in COAD (p = 0.02), LGG (p = 0.03), LIHC (p = 4.50E−04) and PRAD (p = 1.70E−04) patients with high expression. In ACC (p = 5.40E−03), LGG (p = 7.10E−08), LIHC (p = 5.80E−05), MESO (p = 0.02) and PRAD (p = 3.40E−08), high HAUS5 expression was significantly associated with shorter progression-free interval (PFI) (Fig. 2D). From the results, we found that HAUS5 was significantly associated with all four survival indicators in LIHC, LGG and PRAD, which suggests that HAUS5 plays an important role in a variety of tumors.

### High expression and clinical correlation of HAUS5 in LIHC

**Figure 3:**
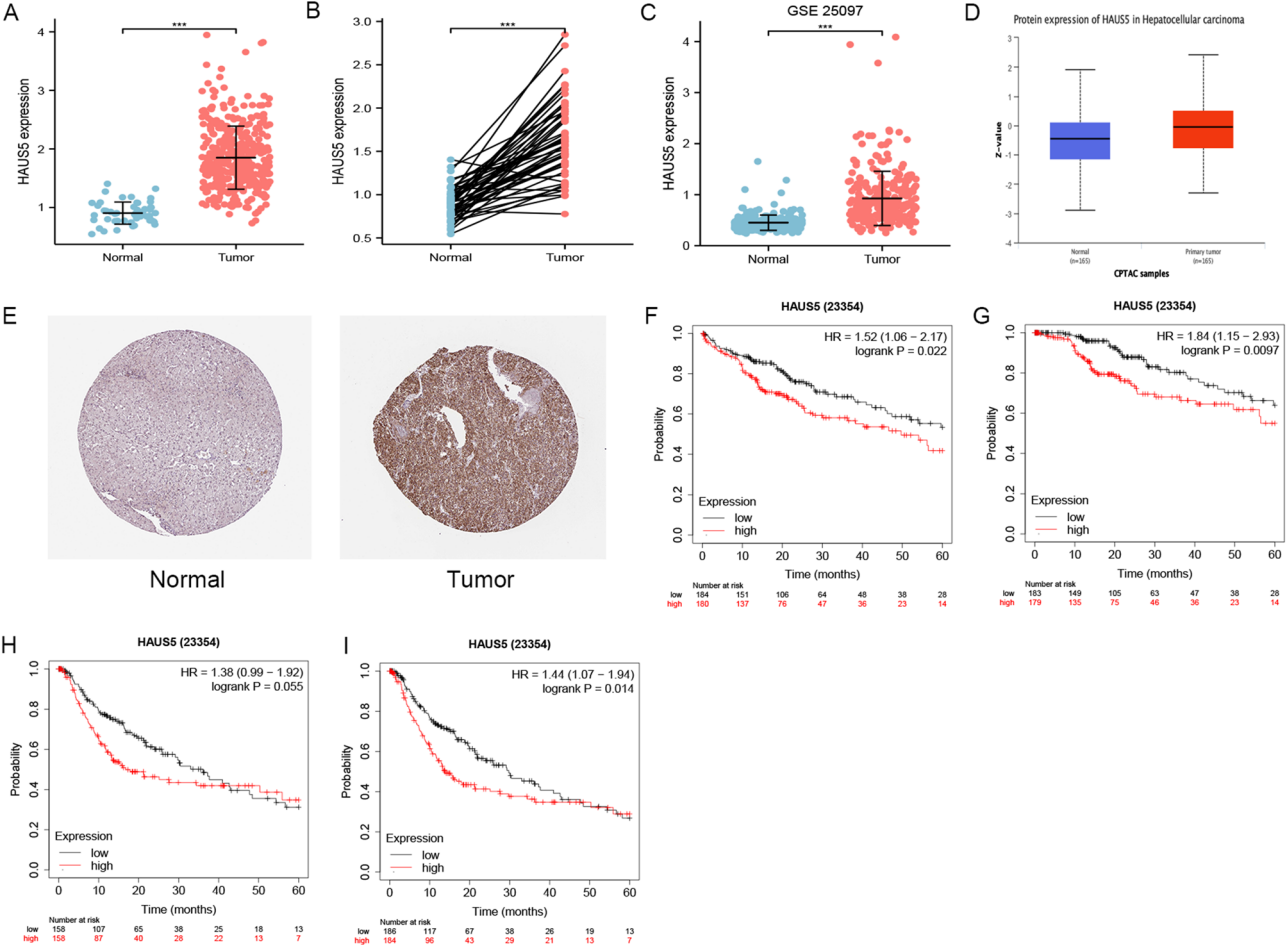
High expression and prognostic value of HAUS5 in LIHC. HAUS5 was overexpressed in (**A**) TCGA-LIHC samples, (**B**) TCGA-LIHC paired samples and (**C**) the GSE25097 dataset. (**D**) The protein expression levels of HAUS5 based on CPTAC. (**E**) The protein levels of HAUS5 analyzed by the Human Protein Atlas. HAUS5 expression was associated with prognosis according to the Kaplan‒Meier Plotter database: (**F**) OS, (**G**) DSS, (**H**) RFS and (**I**) PFS. * P < 0.05, ** P < 0.01, *** P < 0.001.

We conducted bioinformatics analysis using R packages with 419 samples from the TCGA database to verify the findings in LIHC across cancers. According to Fig. 3A, we found that HAUS5 mRNA expression was significantly higher in LIHC samples (369 cases) than in normal samples (50 cases), which was consistent with the pan-cancer analysis. Figure 3B showed that HAUS5 mRNA expression was higher than that in the cancer adjacent tissues (n = 50). Figure 3C showed that GSE25097 dataset analysis validated differential HAUS5 expression. The protein expression level of HAUS5 in normal and tumor tissues was found by using clinical specimens from the CPTAC and HPA databases. According to Fig. 3D,E, HAUS5 was relatively highly expressed in the tumor tissues compared to the normal tissue, similar to the mRNA results. We examined the relationships between HAUS5 mRNA expression and the prognosis of LIHC patients via Kaplan–Meier survival curves to explore whether HAUS5 could be used as a prognostic biomarker in LIHC. The findings indicated that patients with high HAUS5 expression had poorer prognosis than those with low expression in LIHC cohort (OS: cutoff value = 513, (hazard ratio) HR = 1.52, p = 0.022; DSS: cutoff value = 513, HR = 1.84, p = 0.0097; Relapse-free survival (RFS): cutoff value = 520, HR = 1.38, p = 0.055; Progression-free survival (PFS): cutoff value = 513, HR = 1.44, p = 0.014) (Fig. 3F–I).

**Figure 4:**
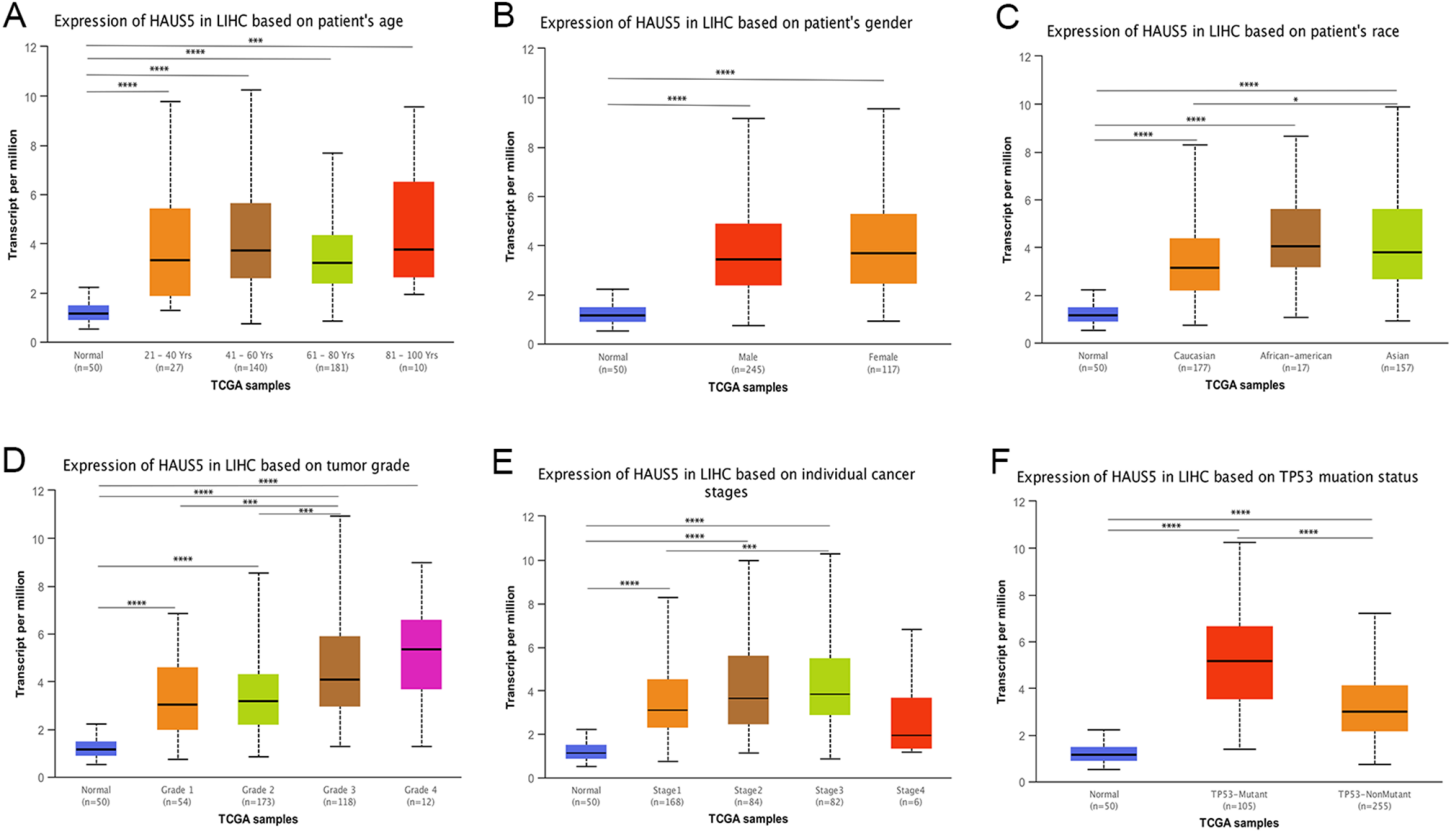
Association between the expression level of HAUS5 and clinical features in LIHC analyzed via the UALCAN database. HAUS5 differential expression in liver cancer patients according to the characteristics of age (**A**), sex (**B**), race (**C**), tumor grade (**D**), cancer stage (**E**) and TP53 mutation status (**F**). *P < 0.05, **P < 0.01, ***P < 0.001, ****P < 0.0001.

To determine the significance of HAUS5 expression in tumor development, we observed the correlation between HAUS5 expression and pathological features of LIHC in the Kaplan‒Meier Plotter database (Table 1). Upregulated HAUS5 expression was linked with poorer OS and PFS in male patients (OS HR = 1.68, p = 0.023; PFS HR = 1.69, p = 0.0041) and female patients (OS HR = 2.38, P = 0.0043). Specifically, increased HAUS5 mRNA expression was associated with poorer OS and PFS in stage I + II (OS HR = 2.63, p = 6.20E-05; PFS HR = 1.64, p = 0.014) LIHC patients. Furthermore, we discovered that OS and PFS in patients with grade 2 (OS HR = 2.13, p = 0.0053; PFS HR = 2.02, p = 0.0051) were related to HAUS5 expression. HAUS5 was related to poorer OS and PFS in patients without hepatitis virus infection (OS HR = 1.73, p = 0.023; PFS HR = 2.75, p = 2.70E−05). Subsequently, we explored the differences in HAUS5 expression in normal and hepatocellular carcinoma tissues according to different clinical characteristics, such as age, sex, race, tumor grade, TNM stage and TP53 mutation status, in using the UALCAN database. As shown in Fig. 4A–F, our results indicated that HAUS5 expression was closely related to the clinical parameters and development of liver cancer.

**Table 1:** Correlation between the HAUS5 expression and pathological features of LIHC in OS and PFS.

Clinicopathological characteristics	Overall survival (n = 364)			Progression-free survival (n = 370)		
	N	Hazard ratio	p	N	Hazard ratio	p
Gender						
Female	118	2.38(1.29–4.39)	***0.0043***	120	1.66(0.97–2.85)	0.061
Male	246	1.68(1.07–2.63)	***0.023***	246	1.69(1.18–2.43)	***0.0041***
TNM stage						
I + II	253	2.63(1.61–4.30)	**6.20E-05**	254	1.64(1.10–2.45)	**0.014**
III + IV	87	0.78(0.43–1.42)	0.420	88	1.61(0.85–3.06)	0.140
Grade						
1	55	0.65(0.24–1.72)	0.380	55	1.89(0.86–4.18)	0.110
2	174	2.13(1.24–3.68)	***0.0053***	175	2.02(1.22–3.32)	***0.0051***
3	118	1.66(0.88–3.12)	0.110	119	1.92(0.98–3.79)	0.054
4	12			12		
Alcohol consumption						
Yes	115	1.95(0.88–4.33)	0.093	115	2.14(1.17–3.93)	***0.012***
No	202	2.03(1.27–3.26)	***0.0028***	204	1.51(0.98–2.34)	0.061
Hepatitis virus						
Yes	150	2.55(1.32–4.95)	**0.0041**	152	0.85(0.50–1.43)	0.530
No	167	1.73(1.07–2.80)	**0.023**	167	2.75(1.68–4.49)	***2.70E-05***
Vascular invasion						
None	203	1.73(1.00–3.01)	***0.049***	204	1.33(0.84–2.10)	0.220
Micro	90	2.07(0.96–4.45)	0.058	91	1.72(0.96–3.08)	0.063
Macro	16					

Bold values indicate p < 0.05.

### Construction of a nomogram to predict the prognosis of LIHC patients

**Figure 5:**
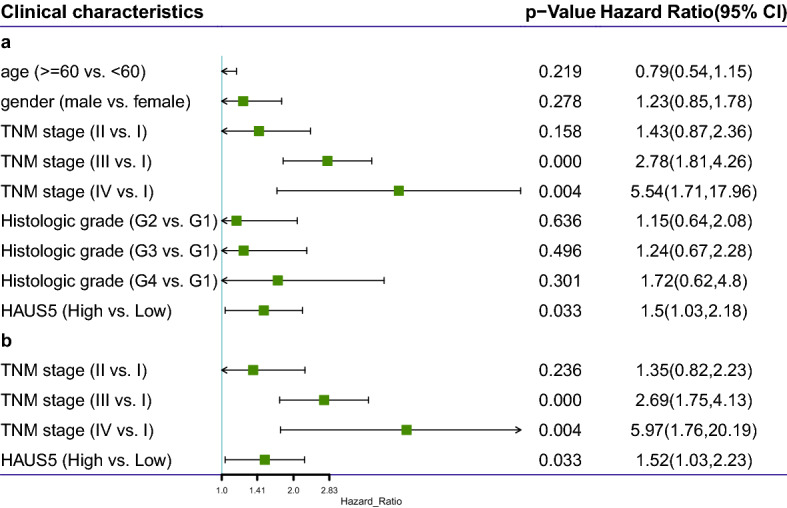
Univariate (a) and multivariate (b) Cox regression analyses of the correlations of HAUS5 and other clinical parameters with OS in TCGA-LIHC cohorts.

**Figure 6:**
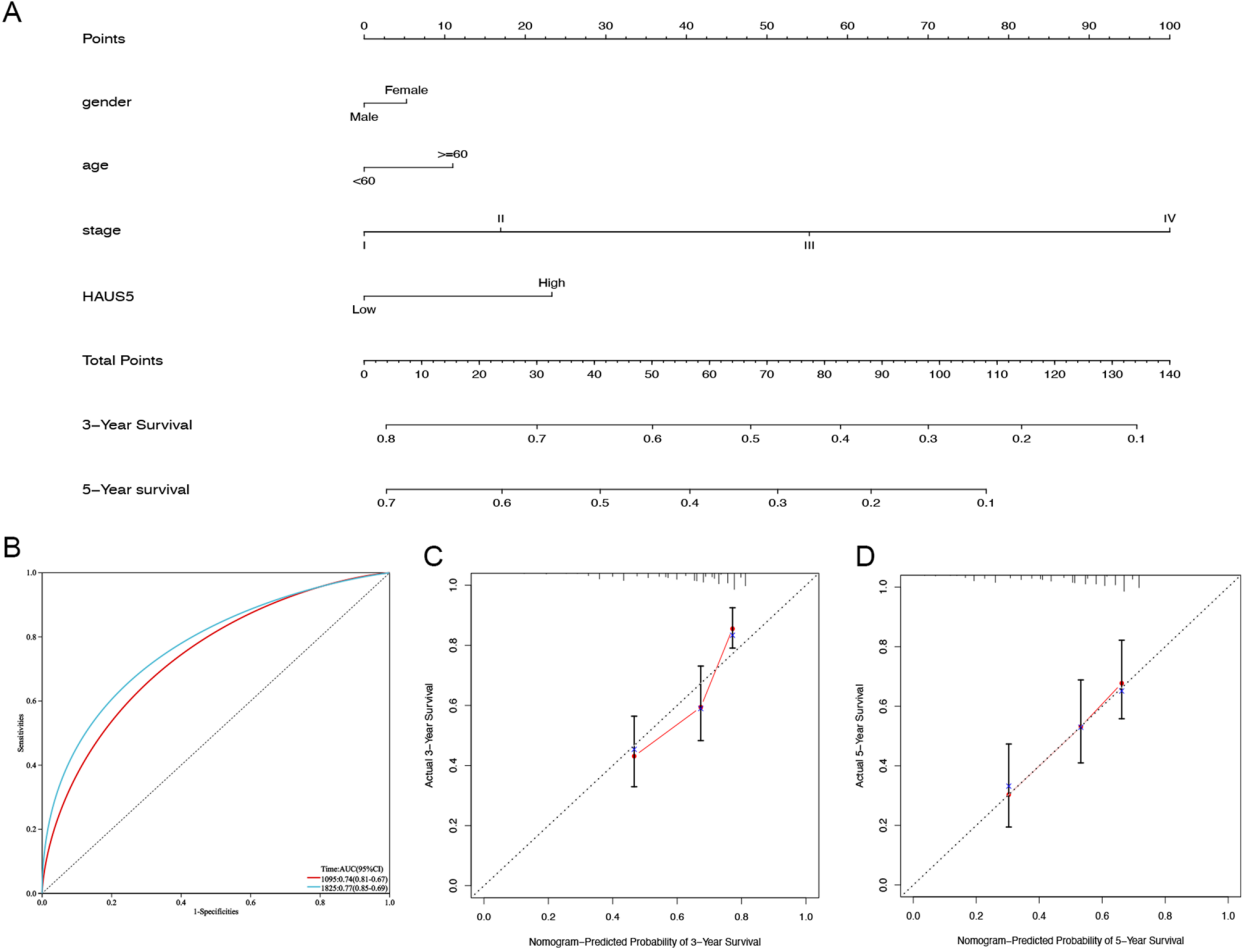
Construction and evaluation of a nomogram to predict 3- and 5-year OS in LIHC patients. (**A**) The nomogram was applied to predict the 3- and 5-year OS of LIHC patients, and the total score on the bottom scale implies the probability of OS. (**B**) ROC curves to evaluate the nomogram accuracy for predicting 3- and 5-year OS in LIHC patients. (**C**,**D**) Calibration curves of the nomogram for the prediction of survival rates at 3 and 5 years.

According to the OS univariate and multivariate Cox regression analyses (Table 2), TNM stage and HAUS5 expression were significantly identified (Fig. 5). A nomogram integrating age, sex, TNM stage and HAUS5 risk score was constructed (Fig. 6A). Total points were calculated by adding the points of the genetic score, age, sex and TNM stage. The calibration curves for predicting 3- and 5-year OS indicated that the nomogram-predicted survival closely corresponded with actual survival outcomes. Based on the TCGA cohort, the 3-year nomogram’s AUC was 0.74, and the 5-year nomogram’s AUC was 0.77 (Fig. 6B). The almost overlapping reference lines indicated that the model was accurate (Fig. 6C,D).

**Table 2:** a. Association with overall survival and clinicopathologic characteristic in LIHC patients using univariate cox regression. b. Multivariate survival analysis using cox regression.

Clinical characteristics	p-value	HR (95%CI)	Lower	Upper
a				
Age (≥ 60 vs < 60)	0.219	0.79 (0.54–1.15)	0.54	1.15
Gender (male vs female)	0.278	1.23 (0.85–1.79)	0.85	1.79
TNM stage (II vs. I)	0.158	1.43 (0.87–2.36)	0.87	2.36
TNM stage (III vs. I)	**0.00E+00**	2.78 (1.81–4.26)	1.81	4.26
TNM stage (IV vs. I)	**0.004**	5.54 (1.71–17.96)	1.71	17.96
Histologic grade (G2 vs. G1)	0.636	1.15 (0.64–2.08)	0.64	2.08
Histologic grade (G3 vs. G1)	0.496	1.24 (0.67–2.28)	0.67	2.28
Histologic grade (G4 vs. G1)	0.301	1.72 (0.62–4.80)	0.62	4.80
HAUS5 (high vs low)	**0.033**	1.50 (1.03–2.12)	1.03	2.12
b				
Age (≥ 60 vs < 60)	0.315	1.22 (0.83–1.79)	0.83	1.79
Gender (male vs female)	0.64	0.91 (0.61–1.35)	0.61	1.35
TNM stage (II vs. I)	0.236	1.35 (0.82–2.24)	0.82	2.24
TNM stage (III vs. I)	**0.00E+00**	2.69 (1.75–4.13)	1.75	4.13
TNM stage (IV vs. I)	**0.004**	5.97 (1.76–20.19)	1.76	20.19
HAUS5 (high vs low)	**0.033**	1.52 (1.03–2.23)	1.03	2.23

Bold values indicate p < 0.05.

### Co-expressed genes and functional enrichment analysis

**Figure 7:**
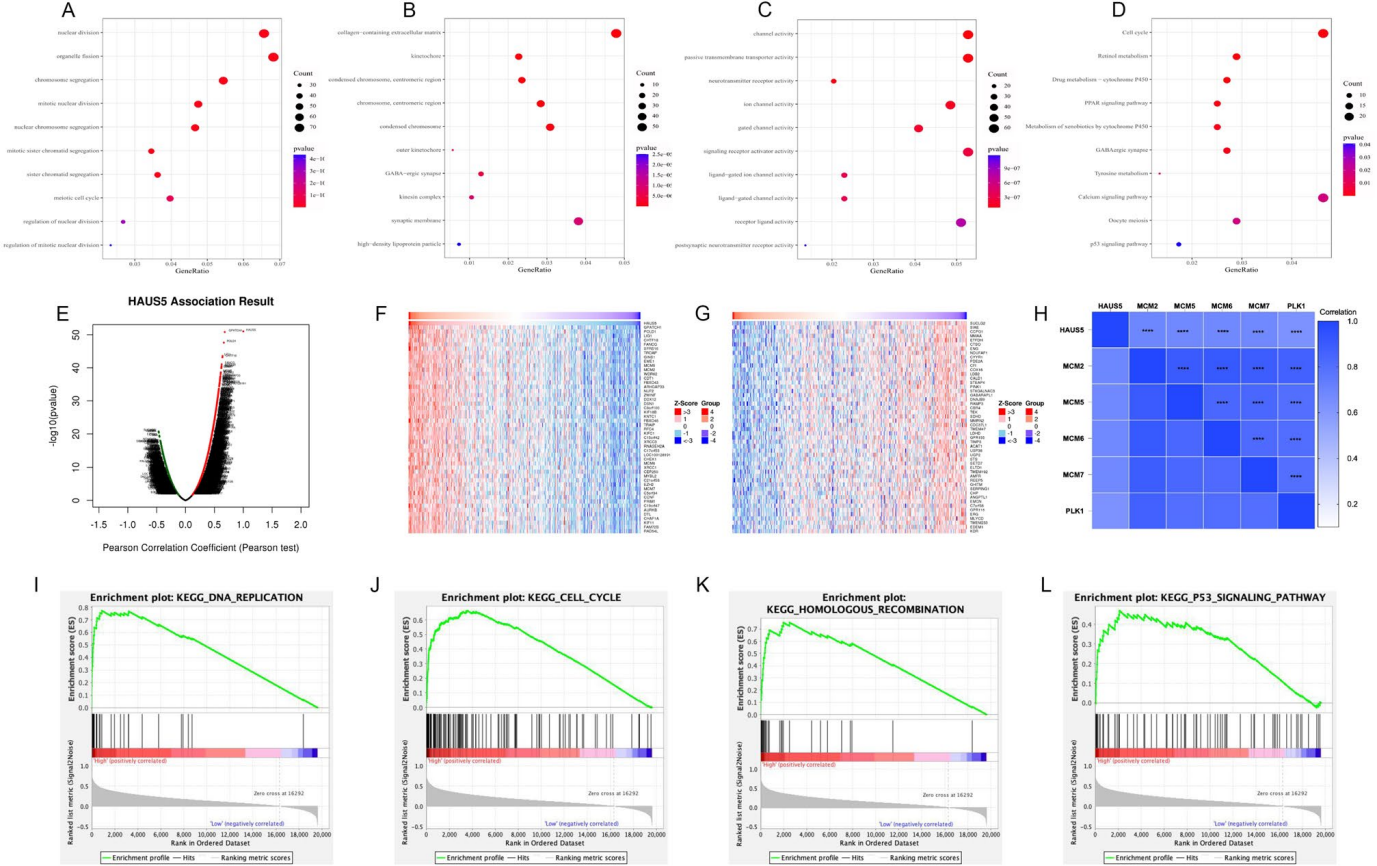
Functional enrichment analysis and co-expressed genes of HAUS5. GO function enrichment analysis based on three aspects, including (**A**) BPs, (**B**) CCs, and (**C**) MFs. (**D**) KEGG pathway enrichment analysis (Sourced from www.kegg.jp/kegg/kegg1.html). HAUS5 co-expressed genes in LIHC analyzed by the LinkedOmics database. (**E**) Volcano map of genes highly correlated with HAUS5 according to Pearson’s test in the LIHC cohort. (**F**,**G**) Heatmaps show the top 50 positively correlated genes and negatively correlated genes of HAUS5 in LIHC datasets. (**H**) Heatmap of the relationship between HAUS5 and cell cycle-related genes. GSEA showed pathways enriched in (**I**) DNA replication, (**J**) the cell cycle, (**K**) homologous recombination and the (**L**) P53 signaling pathway. *P < 0.05, **P < 0.01, ***P < 0.001, ****P < 0.0001.

Based on the TCGA-LIHC dataset, we examined the differentially expressed genes (DEGs) between the low- and high-expression groups to investigate the biological functions of HAUS5. The enriched biological pathways (BP) terms were associated with development, including “nuclear division”, “organelle fission”, “chromosome segregation”, “mitotic nuclear division” and “nuclear chromosome segregation” (Fig. 7A). The enriched cellular components (CC) terms were related to “collagen-containing extracellular matrix”, “synaptic membrane”, “condensed chromosome”, “chromosome, centromeric region” and “condensed chromosome, centromeric region” (Fig. 7B). For molecular function (MF), DEGs were mainly enriched in “channel activity”, “passive transmembrane transporter activity”, “signaling receptor activator activity”, “receptor ligand activity” and “ion channel activity” (Fig. 7C). We also performed KEGG pathway enrichment analysis, which showed that high HAUS5 expression was mainly related to the cell cycle, retinol metabolism, drug metabolism-cytochrome P450, PPAR and P53 signaling pathways (Fig. 7D).

The LinkedOmics database was used to examine HAUS5 co-expressed genes. As shown in the volcano map (Fig. 7E), 4836 genes (dark red dots) had significant positive correlation with HAUS5 and 2730 genes (dark green dots) were negatively related (false discovery rate (FDR < 0.01). Figure 7F,G showed the top 50 genes that were positively correlated and negatively correlated with HAUS5. In addition, we created a heatmap of the correlation between HAUS5 and several genes involved in cell cycle regulation (Fig. 7H). As a potential marker of cell proliferation, MCM2-7 is required for DNA replication, and is related to the progression and prognosis in liver cancer^18,19^.PLK1 is an important cell cycle-regulated protein $kinase that is positively correlated with HAUS5 mRNA expression. As shown in Fig. 7I–L, GSEA showed that genes positively correlated with HAUS5 expression were mainly enriched in DNA replication, the cell cycle, homologous recombination and the P53 signaling pathway. These results suggested a correlation of HAUS5 on DNA replication and cell division.

### HAUS5 expression is associated with immune infiltration

**Figure 8:**
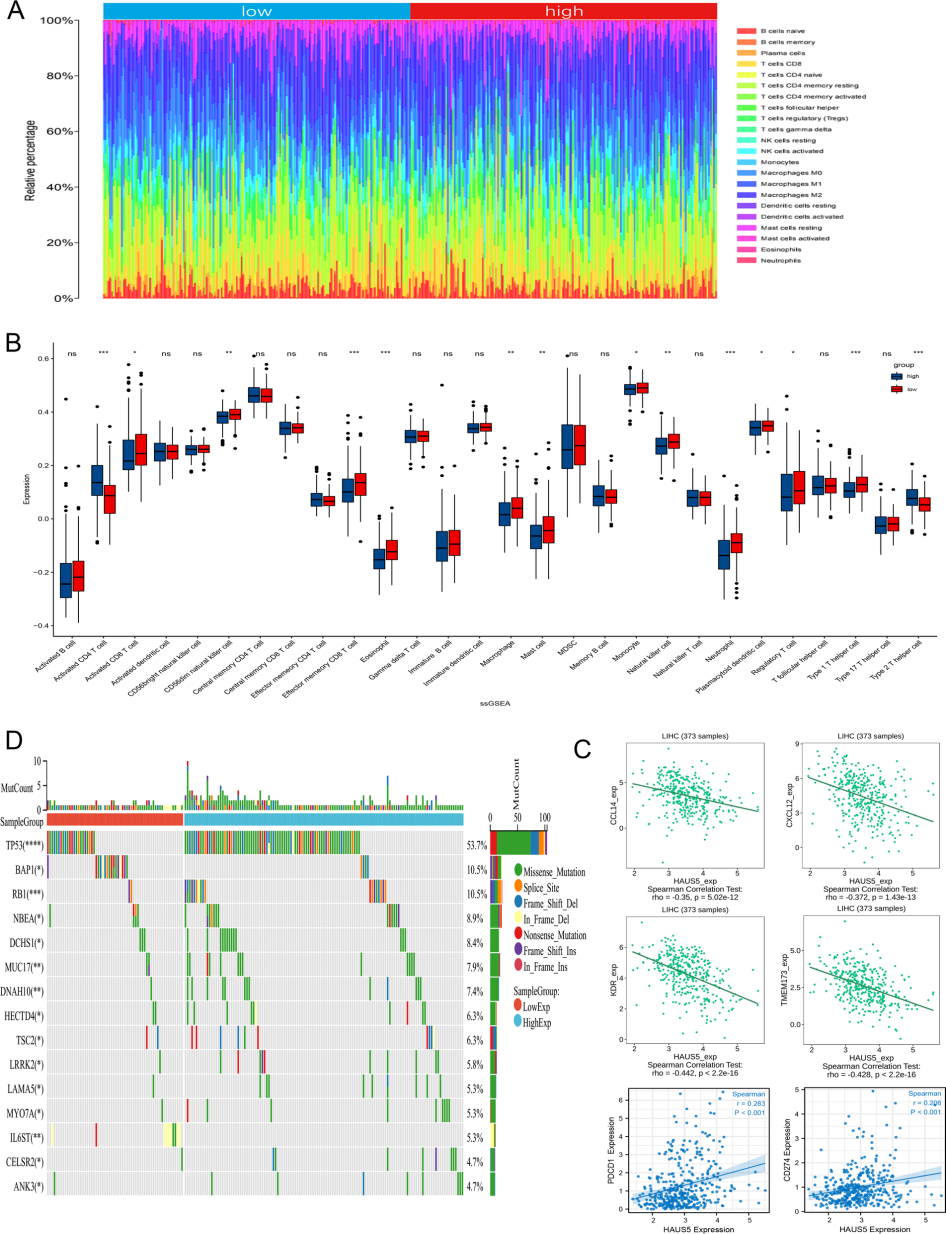
HAUS5 expression was correlated with immune infiltration and mutations in the tumor microenvironment. (**A**) The infiltration of 22 immune cell types in TCGA-LIHC patients. (**B**) The boxplot shows the correlation between HAUS5 expression and 28 immune cell subsets. (**C**) Correlation between HAUS5 expression and immunomodulatory targets. (**D**) The top 15 genes mutation information in liver cancer samples showed in the waterfall plot. *P < 0.05, **P < 0.01, ***P < 0.001. ns, no statistical significance.

The level of immune cell infiltration in liver cancer patients was assessed by applying the CIBERSORT algorithm to the transcriptome of the TCGA-LIHC cohort (Fig. 8A). Subsequently, we divided the samples into groups according to the median expression of HAUS5 and used the ssGSEA method to study the distribution of 28 types of immune cells in different subgroups in liver cancer tissues (Fig. 8B). Twelve immune cell subtypes (activated CD8 T cells, CD56dim natural killer cells, effector memory CD8 T cells, eosinophils, macrophages, mast cells, monocytes, natural killer cells, neutrophils, plasmacytoid dendritic cells, regulatory T cells and type 1 T helper cells) showed higher expression of HAUS5 in low group than that of high group. The results showed that the low HAUS5 expression group had a higher abundance of immune infiltrating cells, especially activated CD8 T cells and natural killer cells. Given the importance of the clinical use of immunotherapy in patients with LIHC, we further explored the correlation of HAUS5 expression and immune checkpoints. From Fig. 8C we found that HAUS5 expression showed varying degrees of correlation with most immunomodulatory targets (CCL14 (r = − 0.35, p = 5.02e−12), CXCL12 (r = − 0.372, p = 1.43e−13), KDR (r = − 0.442, p = 2.2e−16), TMEM173 (r = − 0.428, p = 2.2e−16), PDCD1 (r = 0.283, p < 0.001) and PDL1 (r = 0.206, p < 0.001)). Furthermore, a total of 369 LIHC samples were included in which mutations were detected. In the Fig. 8D of waterfall plot which showed the top 15 mutated genes, 189 (51.2%) LIHC patients had somatic mutations with mutation types indicated by different color-coded annotations. Compared to the low HAUS5 expression group, the high group significantly had more mutations in terms of the top 10 mutated genes in LIHC included TP53, BAP1, RB1, NBEA, DCHS1, MUC17, DNAH10, HECTD4, TSC2 and LRRK2. These results suggested that HAUS5 expression was correlated with immune infiltration in hepatocellular carcinoma.

### Knockdown of HAUS5 shows tumor-suppressive effects

**Figure 9:**
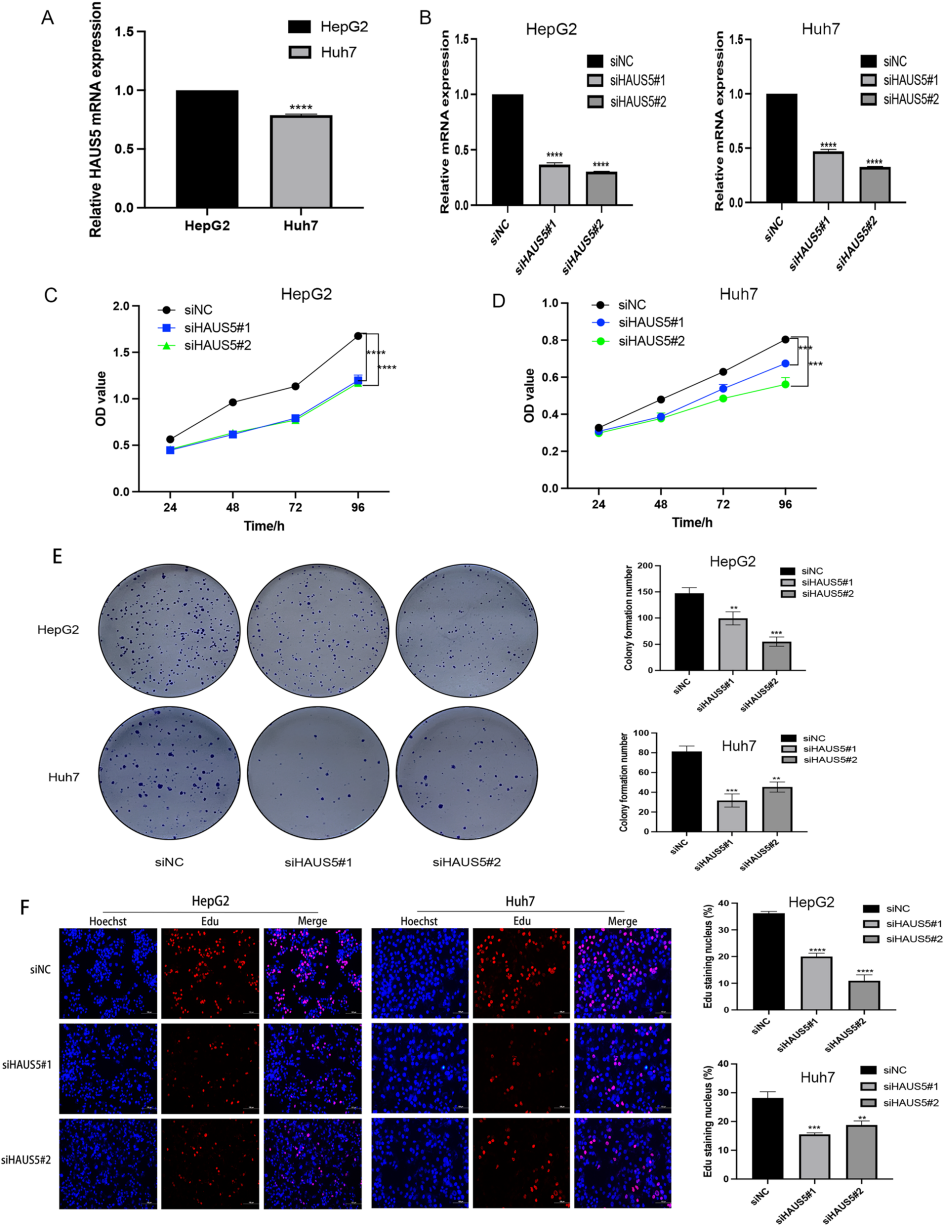
The effect of knockdown of HAUS5 on cell proliferation. (**A**) The level of HAUS5 expression in HepG2 and Huh7. (**B**) qRT‒PCR indicated that HAUS5 expression was decreased after knockdown of HAUS5 in HepG2 and Huh7 cells. (**C**,**D**) CCK-8 assay and (**E**) cell colony formation assay showed that cell proliferation was inhibited. (**F**) EdU assays showed that the capability of DNA replication decreased in cells with HAUS5 knockdown. *P < 0.05, **P < 0.01, ***P < 0.001, ****P < 0.0001.

In in vitro trials, we compared the baseline expression level of HAUS5 in HepG2 and Huh7 cell lines through qRT‒PCR and the result showed that the HAUS5 mRNA expression of HepG2 was higher than that of Huh7 (Fig. 9A). We knocked down HAUS5 expression by using siRNAs in HepG2 and Huh7 cells, which was verified by qRT‒PCR (Fig. 9B). CCK-8 and colony formation assays were used to evaluate the proliferation of LIHC cells. We found that HAUS5 knockdown significantly inhibited cell proliferation (Fig. 9C,D). Cells of different treatment groups were inoculated into 6-well plates. The plates were seeded with 1000 cells (HepG2) or 3000 cells (Huh7). The cells were fixed after 14 days of culture, stained with Giemsa and then counted. The results showed that the colony formation rate was significantly lower in the HAUS5 knockdown group than in the control groups (Fig. 9E). The EdU assay indicated that DNA replication was suppressed in cells with HAUS5 knockdown (Fig. 9F). Our studies indicated that inhibition of HAUS5 expression significantly reduced the proliferation of liver cancer cells.

## Discussion

As a member of the HAUS family, HAUS5 is involved in the formation of the augmin complex^11,20^. In addition, HAUS5 is involved in the formation of microtubules during mitosis and plays an important role in spindle assembly, chromosome segregation and cytokinesis^12,21^. Tubulin-binding compounds that inhibit microtubule dynamics and disrupt mitotic spindle formation have been used to treat various cancers^22^. HAUS5 may will be a new therapeutic target into clinical practice of cancer therapy^16,23^. However, the expression of HAUS5 in LIHC and its significance remain unclear.

In this study, we firstly found that HAUS5 mRNA is highly expressed in most tumors and is closely related to the prognosis of various tumors. The GSE25097 dataset validated the aberrant expression of HAUS5 in HCC. By analyzing the HAUS5 mRNA expression level in LIHC patients grouped by different clinical variables, it was found that its abnormal expression was closely related to age, sex, TNM stage, tumor grade and TP53 mutation. High mRNA expression of HAUS5 was significantly correlated with multiple survival indicators in HCC patients. Based on multivariate Cox regression analysis, a unique nomogram evaluation model was constructed that confirmed the close relationship between risk scores and clinicopathological parameters. These results suggest that HAUS5 may be a novel oncogene of HCC and can be used as a potential biomarker for HCC diagnosis and prognosis evaluation.

To further clarify the biological function of HAUS5, GO function and KEGG pathway enrichment analysis were performed, and GSEA was used to further validate its function. In HCC, HAUS5 plays an important role in regulating DNA replication and the cell cycle, and its high expression is significantly enriched in the p53 signaling pathway. In addition, we analyzed the co-expressed genes of HAUS5. The results showed that HAUS5 was significantly associated with multiple genes involved in cell division and cell cycle regulation, including MCM2^24^, MCM5^25^, MCM6^19^, MCM7^26^ and PLK1^27^, which have been shown to play important roles in a variety of cancers. Previous studies identified PLK1, a mitotic kinase that acts as a regulator in the cell cycle and affects cell cycle progression through the p53 signaling pathway^28,29^. The expression of HAUS5 in HCC tissues was significantly positively correlated with the expression of PLK1, which was supervised by HAUS5 during the G2/M transition. Therefore, we speculate that HAUS5 may affect the occurrence and development of HCC by regulating the expression of the PLK1 and the activation of the p53 signaling pathway. While the main functions of p53 include promoting cell cycle arrest and apoptosis^30^, in a recent study, p53 activation is associated with an increase in the development of liver cancer in human with chronic liver disease^31^. TP53 mutations frequently occur in human cancers, the accumulation of which is considered to be a highly specific marker of malignancy^32,33^. TP53 mutation was closely associated with the immune microenvironment, which resulted in the downregulation of the immune response in HCC^34^. Patients with tumour TP53 mutations have shorter OS and RFS compared to HCC patients with wild-type TP53^35^. In our study, we found that patients with TP53-mutated HCC had the highest expression of HAUS5 compared with patients with TP53-wild type HCC and those without HCC. The HAUS5 expression correlated with the TP53 mutation of liver cancer tumor cells, which deserves further attention and research in future study.

The interaction between the tumor and the immune system plays a key role in the occurrence, progression and treatment of cancer^36,37^. The liver is an immune organ rich in a variety of immunocompetent cells^38^. However, HCC still has a poor prognosis, and new strategies for immunotherapy are needed^39^. In recent years, various treatment regimens for HCC have been clinically applied. Tumor endothelial cells can be altered through the combination of anti-angiogenic drugs and immune checkpoint inhibitors, which leads to the increasing infiltration of effector immune cells and the combination of atezolizumab and bevacizumab has been verified to improve OS of HCC patients in advanced stage which was approved as first-line therapy in 2020^40,41^. We explored the possible role of HAUS5 in HCC immunotherapy by ssGSEA, and we found that the expression of HAUS5 was significantly associated with the levels of most tumor-infiltrating immune cells, especially activated CD8 T cells and natural killer cells. Cytotoxic T lymphocytes (CTLs) and natural killer cells are antitumor immune cells that play an important role in resisting tumor progression, and CD8 + T lymphocytes are the main effector cell subsets in HCC^42,43^. In this study, the level of infiltrating activated CD8 + T cells and natural killer cells in the low group of HAUS5 expression was higher than that in the high-expression group, so tumor progression was possibly suppressed in low expression group. Furthermore, immune checkpoint blockade is an irreplaceable type of cancer immunotherapy. Immune checkpoint inhibitors have emerged as a potentially effective treatment for patients with advanced HCC^44^. We found that HAUS5 expression was significantly negatively correlated with the expression of multiple immunomodulatory targets. The PD1/PDL1 signal transduction pathway is an important factor in tumor immunosuppression and can inhibit the excitation of T lymphocytes and enhance the immune tolerance of tumor cells, thereby facilitating tumor immune escape^45^. HAUS5 expression is positively correlated with PD1 and PDL1 expression, and the binding of PD1 to PDL1 suppresses T-cell-mediated immune surveillance, leading to loss of the immune response and even T-cell apoptosis^46^. PD1 and PDL1 expression are positively associated with HAUS5 expression which possibly leads to the poor prognosis of LIHC patients with high HAUS5 expression. Absolutely, this is exactly what we need further experiments to investigate in future studies.

To further verify the role of HAUS5 in HCC, we performed in vitro experiments, including CCK-8, cell colony formation and EdU assays. The results showed that knockdown of HAUS5 expression could inhibit DNA replication, thereby attenuating the proliferation and colony formation of HCC cells. Since the levels of HAUS5 in Huh7 are lower than that of HepG2, this could explain why the knockdown had a greater effect on the growth of Huh7. Considering there are different subtypes of HCC in patients and our in vitro results demonstrate the baseline levels may drive the effect and role we are proposing of HUAS5 in HCC, the combination of our data supports stratification of patients to low and high to further investigate the role and function of HAUS5 in HCC. In conclusion, the above results revealed firstly that HAUS5 may be a potential diagnostic and prognostic biomarker for HCC and possibly related to mutation and immune infiltration in HCC patients. However, the study still has some limitations. Most of our results are limited to data mining analysis results, and we need more in vitro and in vivo experiments to investigate the exact mechanism of HAUS5 in LIHC.

## Conclusions

In this study, the pan-oncogene HAUS5 was identified as a novel prognostic marker for liver cancer by bioinformatics analysis. We further verified by in vitro experiments that inhibition of HAUS5 expression suppressed the proliferation of liver cancer cells. The identification of novel biomarkers promises to improve patient survival. However, this study has some limitations. Most of our findings are based on data analysis, and more experiments are needed to further verify and explore potential mechanisms in the future.

## Materials and methods

### Data collection and analysis

Gene expression data of 424 samples (50 normal samples and 374 liver cancer samples), clinical information of the corresponding TCGA-LIHC patients and data for 31 normal tissues from GTEx were downloaded based on the University of California, Santa Cruz (UCSC) Xena database (https://xenabrowser.net/datapages/). Cases with insufficient data were excluded from further processing. HAUS5 expression data of tumor cell lines were obtained from the CCLE database (https://sites.broadinstitute.org/ccle/datasets). Dataset GSE25097 includes information on 268 patient samples and 249 normal samples, which was downloaded from the GEO database (https://www.ncbi.nlm.nih.gov/geo/). HAUS5 gene expression data from these downloaded datasets were extracted and plotted in a data matrix for further analysis via R software (Version 4.1.1), and tumor samples were divided into low and high expression groups according to the median expression of HAUS5 for further study. Below are the online tools that were used.

### Pan-cancer prognosis analysis

A pan-cancer analysis of the TCGA project was based on Sangerbox (http://sangerbox.com/), which is a free online data analysis platform. OS, DSS, DFI and PFI were analyzed across all 33 types of cancers and were shown by forest plots. Statistical analysis with the log-rank test was conducted to obtain prognostic significance.

### Kaplan‒Meier plotter database analysis

The Kaplan‒Meier Plotter database (http://kmplot.com/analysis/) allows the comparison of survival in patients with different cancers grouped by mRNA expression levels. Our study investigated whether the expression of HAUS5 was associated with the prognosis of LIHC patients obtained from the TCGA in terms of OS, DSS, RFS, and PFS using Kaplan‒Meier Plotter. Then, Kaplan–Meier curves were drawn for patients divided into two groups based on the optimal cutoff of HAUS5 expression. The logrank p value was calculated as well as the HR with 95% confidence intervals.

### UALCAN database analysis

The UALCAN database (http://ualcan.path.uab.edu) collected RNA-seq and clinical data of multiple cancer types from TCGA dataset and offered a useful platform to analyze gene expression between tumor and normal tissues. This database was used to analyze the relationship between HAUS5 mRNA expression and clinicopathological features in TCGA-LIHC cohort consists of 50 normal samples and 371 tumor samples. Furthermore, the protein level expression of HAUS5 was studied through the CPTAC model and HPA database (http://www.proteinatlas.org/), which is a convenient website providing immunohistochemistry-based expression profiles for most cancers. The significance of the differences was estimated by Student’s t test.

### Nomogram built on independent prognostic roles

To investigate whether the HAUS5 gene could predict prognosis independent of other clinical parameters, including age, sex, TNM stage and tumor grade, univariate and multivariate analyses were performed through Cox regression analysis using R packages, and P value < 0.05 was considered statistically significant. Nomograms are widely used to predict cancer prognosis. Clinical features such as age, sex and independent prognostic factors identified by multivariate Cox regression analysis were included to build a nomogram to explore the probability of 3- and 5-year OS for LIHC patients. Moreover, validation of the nomogram was performed by generating calibration curves by a bootstrap method with 1000 resamples. Curves were drawn to compare the nomogram prediction probability against the observed rates. The AUCs of the ROC curves were calculated to assess the discrimination ability of the nomogram.

### GO function and KEGG pathway enrichment analysis

With the DESeq2 R package^47^, we identified DEGs between the low and high HAUS5 expression groups. Utilizing the org.Hs.eg.db (http://www.bioconductor.org/packages/org.Hs.eg.db/) and clusterProfiler^48^ R packages, we conducted GO function and KEGG^49^ pathway enrichment analysis to demonstrate the similarities and differences between the two groups in the BP, CC and MF categories based on GO function enrichment analysis. KEGG pathway enrichment analysis revealed enriched pathways. To be identified as statistically significant, enrichment results had to meet the following criterion: p value < 0.05.

### LinkedOmics database analysis and GSEA

As a multiomics data analysis platform includes data from all 32 TCGA Cancer types and 10 CPTAC cancer cohorts, the LinkedOmics database (http://www.linkedomics.org/login.php) provides us the opportunity to explore and visualize gene expression profiles. With the help of LinkedOmics, we determined the co-expressed genes of HAUS5 in the TCGA-LIHC patient cohort using Pearson's correlation coefficients and displayed the results as heatmaps and volcano maps. According to the median expression of HAUS5, LIHC samples were separated into low and high HAUS5 expression groups. Then, GSEA was performed using GSEA software (v.4.2.3), and enrichment was estimated using the normalized enrichment score (NES). The annotated gene set c2.cp.kegg.v7.5.symbols.gmt was used as the reference gene set, and the significance of the enrichment was assessed with p value < 0.05 and FDR < 0.25 levels.

### Immune infiltration analysis

The proportions of tumor-infiltrating immune cells in 369 TCGA-LIHC samples were calculated using the CIBERSORT^50^ computational method. We also calculated the correlations with Wilcoxon test between the levels of 28 tumor-infiltrating immune cell types and the expression level of HAUS5 by ssGSEA^51^. The database TISIDB (http://cis.hku.hk/TISIDB/index.php) is an online web for tumor and immune system interactions that would become a valuable resource for cancer immunology research and therapy. In our study, we used the TISIDB database with LIHC sample data from TCGA database to study the relationship between HAUS5 expression and chemokines and receptors which was analyzed using Spearman’s test with a significance threshold of p value < 0.05. Additionally, the mutation data of the 369 LIHC samples were downloaded from TCGA database and the Chi-squared test was used to assess differences in the frequency of mutations in each set of samples.

### Cell culture and transfection

Human liver cancer cells (HepG2 and Huh7) were obtained from the Chinese Cell Bank (Shanghai, China) and cultured in DMEM (HyClone, Thermo Fisher Scientific, Waltham, MA, USA) supplemented with 10% fetal bovine serum and 100 U/ml penicillin‒streptomycin at 37 °C and 5% CO2. Three different small interfering RNAs (siRNAs) for the inhibition of HAUS5 expression and a negative control siRNA were purchased from RiboBio Company (Shanghai, China). The sequences of siRNAs targeting HAUS5 were as follows: siRNA-HAUS5-001: 5′-GGGATCTACTCCACATGAA-3′; siRNA-HAUS5-002: 5′-CCTACATCTTGCAGCATGT-3′. HepG2 and Huh7 cells were infected with Lipofectamine 3000 (Invitrogen, Grand Island, NY, USA) for 48 h and subsequently harvested. The HAUS5 knockdown cell lines were validated using quantitative real-time PCR (qRT‒PCR). The cells were infected with siNC, siHAUS5#1 and siHAUS5#2 to achieve HAUS5 downregulation and used for the relevant experiments.

### RNA extraction and qRT‒PCR analysis

Total RNA from approximately 1 × 10^6^ cells was isolated using TRIzol reagent (Invitrogen, CA, USA) according to the manufacturer’s protocol. The sequences of the primers used for qRT‒PCR, including HAUS5 and GAPDH, were as follows: HAUS5, forward-GTCCTGCGTGATGTCCGAA and reverse-ACTGCTGGTACGAAGTGCCAA; GAPDH, forward-TGAAGGTCGGAGTCAACGGATTTGGT and reverse- CATGTGGGCCATGAGGTCCACCAC. The qRT-PCR parameters were 95 °C for 30 s and 95 °C for 5 s, 60 °C for 30 s × 40 amplification cycles. Expression levels were normalized to those of the controls and quantified according to the 2^−△△CT^ method.

### Cell counting kit-8 (CCK-8) and colony formation assays

Cell proliferation capacity was studied with Cell Counting Kit‐8 purchased from MedChemExpress. Cells were seeded at a density of 3 × 10^3^ cells/well in 100 μL of medium into 96‐well microplates (Corning, NY, USA). After cell culture for 24, 48, 72 and 96 h, 10 μL CCK‐8 reagent was added to each well and cultured for 1 h. The absorbance was analyzed at 450 nm using a microplate reader (Thermo, MA, USA). The proliferation of cells was expressed by the absorbance. HepG2 (1000 cells/well) and Huh7 (3000 cells/well) cells were seeded into 6-well plates. After adhesion, the cells were cultured at 37 °C for 14 days. The cells were fixed with methanol for 15 min and stained with crystal violet (0.1%, 15 min), and the numbers of colonies with > 50 cells were counted. All experiments were repeated at least three times.

### 5‐Ethynyl‐2′‐deoxyuridine (EdU) assay

After being infected with siRNA for 24 h, the cells were seeded into 96-well plates (1 × 104 cells/well) and incubated for 24 h before EdU (RiboBio, China) was added. According to the protocol, the cells were then incubated at 37 °C for 3 h and fixed in 4% formaldehyde for 30 min, followed by the addition of 100 µl of 2 mg/ml glycine for 5 min. The cells were permeabilized with 0.5% Triton X-100 for 10 min at room temperature. The cells were washed with PBS, 1× ApolloR reaction mix (100 μL/well) was added, and the cells were reacted with EdU for 30 min at room temperature in the dark. Subsequently, Hoechst 33342 (100 μL/well) was added for 30 min to visualize nuclei. After washing with PBS, positive cells were observed by fluorescence microscopy (DM IL LED, Leica, Wetzlar, Germany).

### Statistical analysis

Data are expressed as the mean ± standard deviation. Student's t test was used to analyze differences between groups. In the in vitro experiments, at least three replicates were conducted. Statistical analysis was performed using GraphPad Prism version 9.1.1 software and R version 4.1.1. The differences were considered significant at p < 0.05 (*), p < 0.01 (**), p < 0.001 (***), and p < 0.0001 (****).

## Data Availability

Expression matrix data were obtained from UCSC Xena (https://xenabrowser.net/datapages/), CCLE (https://sites.broadinstitute.org/ccle/datasets) and GEO database (https://www.ncbi.nlm.nih.gov/geo/), which are publicly available. More information can be accessed from correspondence authors.

## Abbreviations

LIHC: Liver hepatocellular carcinoma
HAUS5: HAUS augmin-like complex subunit 5
TCGA: The Cancer Genome Atlas
GTEx: Genotype-Tissue Expression
CCLE: Cancer Cell Line Encyclopedia
GEO: Gene Expression Omnibus
ssGSEA: Single-sample gene set enrichment analysis
CPTAC: Clinical Proteomic Tumor Analysis Consortium
HPA: Human Protein Atlas
AUC: Area under the curve
OS: Overall survival
HCC: Hepatocellular carcinoma
γ-TuRC: γ-Tubulin ring complex
GO: Gene Ontology
KEGG: Kyoto Encyclopedia of Genes and Genomes
GBM: Glioblastoma
LGG: Brain lower grade glioma
LUAD: Lung adenocarcinoma
COAD: Colon adenocarcinoma
BRCA: Breast invasive carcinoma
ESCA: Esophageal carcinoma
KIRP: Kidney renal papillary cell carcinoma
STAD: Stomach adenocarcinoma
PRAD: Prostate adenocarcinoma
UCEC: Uterine corpus endometrial carcinoma
HNSC: Head and neck squamous cell carcinoma
KIRC: Kidney renal clear cell carcinoma
LUSC: Lung squamous cell carcinoma
THCA: Thyroid carcinoma
READ: Rectum adenocarcinoma
BLCA: Bladder urothelial carcinoma
CHOL: Cholangiocarcinoma
CESC: Cervical squamous cell carcinoma
PAAD: Pancreatic adenocarcinoma
LAML: Acute myeloid leukemia
SKCM: Skin cutaneous melanoma
OV: Ovarian serous cystadenocarcinoma
TGCT: Testicular germ cell tumors
UCS: Uterine carcinosarcoma
ACC: Adrenocortical carcinoma
SARC: Sarcoma
THYM: Thymoma
DSS: Disease-specific survival
MESO: Mesothelioma
DFI: Disease-free interval
PFI: Progression-free interval
ROC: Receiver operating characteristic
HR: Hazard ratio
RFS: Relapse-free survival
PFS: Progression-free survival
DEGs: Differentially expressed genes
BP: Biological pathways
CC: Cellular components
MF: Molecular function
GO: Gene ontology
FDR: False discovery rate
CTLs: Cytotoxic T lymphocytes
UCSC: University of California Santa Cruz
NES: Normalized enrichment score
qRT-PCR: Quantitative real-time PCR
